# A Novel 2-DOF Lorentz Force Actuator for the Modular Magnetic Suspension Platform

**DOI:** 10.3390/s20164365

**Published:** 2020-08-05

**Authors:** Fei Yang, Yong Zhao, Xingke Mu, Wenqiao Zhang, Lingtong Jiang, Honghao Yue, Rongqiang Liu

**Affiliations:** 1 School of Mechatronics Engineering, Harbin Institute of Technology, Harbin 150080, China; yangf@hit.edu.cn (F.Y.); yuehonghao@hit.edu.cn (H.Y.); liurq@hit.edu.cn (R.L.); 2 China Academy of Launch Vehicle Technology Research and Development Center, Beijing 100076, China; muxk@calt.casc (X.M.); jlt_007@163.com (L.J.); 3 Innovation Academy for Microsatellites of CAS, Shanghai 201210, China; zhangwq@microsate.com

**Keywords:** modular magnetic suspension platform, Lorentz force actuator, equivalent magnetic circuit method, distribution of the magnetic field, characteristic of electromagnetic force

## Abstract

The modular magnetic suspension platform depends on multi degree of freedom of Lorentz force actuators for large bearing capacity, high precision positioning and structure miniaturization. To achieve the integration of vertical driving force and horizontal driving force, a novel 2- (two degrees-of-freedom) DOF Lorentz force actuator is developed by designing the pose of the windings and permanent magnets (PMs). The structure and the working principle are introduced. The electromagnetic force mathematical model is established by the equivalent magnetic circuit method to analyze the coupling of magnetic flux. The distribution characteristics of magnetic flux density are analyzed by the finite-element method (FEM). It is found that the coupling of the magnetic flux and the large magnetic field gradient severely reduce the uniformity of the air-gap magnetic field. The electromagnetic force characteristic is investigated by FEM and measurement experiments. The difference between FEM and experiment results is within 10%. The reasons of driving force fluctuation are explained based on the distribution of air-gap magnetic field. The actuator performance are compared under the sliding mode control algorithm and PID control algorithm and the positioning accuracy is 20 μm and 15 μm respectively. Compared with the similar configuration, the motion range and force coefficient of the Lorentz force actuator in this paper are larger. It has a certain guiding significance on the structure design of the multi degree of freed Lorentz force actuator.

## 1. Introduction

The multi-degree-of-freedom positioning platforms are widely used in aerospace and industrial manufacturing, such as optical imaging systems, lithographic processing of semiconductors and precision machining [1,2,3,4,5]. But there are some disadvantages of the friction, wear creep and contact fatigue in mechanical contact platforms, which limits the improvement of positioning accuracy [6,7]. The magnetic suspension platform with electromagnetic actuators can realize the contactless suspension and overcome disadvantages of mechanical contact platforms [8,9]. According to the driving distance, the magnetically levitated actuators can be divided into the long-stroke actuators [10,11,12,13] and the short-stroke actuators [14,15]. The translational motion range of the former in X direction and Y direction is large but the rotation angle is small. The translational motion range of the latter in X direction and Y direction is small but the rotation angle is large, which is more suitable for larger angle attitude adjustment of high precision equipment on spacecraft.

The conventional magnetic suspension platform consists of several single degree of freedom actuators. It is difficult to realize miniaturization for the conventional magnetic suspension platform and it is complicated to optimize the spatial distribution of the single degree of freedom electromagnetic actuators. The modular magnetic suspension platforms have advantages of tight configuration and light weight. And modular design makes it easy to optimize the structure of the electromagnetic-mechanical system [16,17,18,19].

The multi degree of freedom electromagnetic actuators can produce driving force in multiple directions, which are the critical components of the modular magnetic suspension platform [20,21]. According to different driving forces, the magnetically levitated actuators can be divided into the resistance magnetic actuators [22,23,24] and the Lorentz force actuators. Compared with the resistance magnetic actuators, due to the better linearity between the electromagnetic force and the control current, the Lorentz force actuators have many advantages such as no iron loss, high control precision, high bandwidth and so on [25,26]. In Reference [27], a cylindrical Lorentz-force magnetic bearing can output deflection moment but the translational drive cannot be realized. In Reference [28], two groups of circuit board wires are perpendicular to each other in the magnetic field to generate vertical driving force and horizontal driving force. But the effective length of the electrified wires is small and the driving force is low. In Reference [29], the cylindrical windings are arranged in the axial air-gap of cylindrical permanent magnet rings to realize radial driving and axial driving. The movement range is ±1 mm in the radial direction and ±5 mm in the axial direction. Its overall size is 125 mm × 125 mm × 125 mm. In Reference [30], the winding under the permanent magnet (PM) generates vertical driving force and the winding along the side of the PM produces horizontal driving force. The translational motion range of the actuator is ±5 mm but the vertical motion range is only 200 μm. The size of each coil is 40 mm × 40 mm × 17.5 mm. In Reference [31], the rectangular winding and the cylindrical winding are under the cylindrical magnet with axial magnetization. The two windings generate driving forces in the vertical directions and horizontal directions respectively. The overall size is 60 mm × 60 mm × 40 mm and the operation range is 200 μm × 200 μm × 200 μm. However, it has the disadvantages of more magnetic flux leakage and low utilization rate of magnetic field in References [30,31]. The above two degrees-of-freedom (DOF) electromagnetic actuators have the disadvantages of small output force and small moving range. In addition, magnetic field coupling is an important factor to determine driving precision of electromagnetic actuators [32,33,34].

To improve force coefficient and deflection angle and investigate the magnetic field coupling, a novel 2-DOF Lorentz force actuator is developed by designing the pose of the PMs and the windings. It has the advantages of large output force and wider moving range. This paper is organized as follows—The next section will introduce the structure and working principle of the Lorentz force actuator. In Section 3, electromagnetic force model are established by equivalent magnetic circuit method (EMCM) and the coupling of magnetic circuit is analyzed. In Section 4, the distribution characteristics of magnetic field at critical regions are analyzed by finite-element method (FEM). In Section 5, the change rules of electromagnetic force are analyzed by FEM and experiments. The reasons for the fluctuation of driving force are analyzed based on the distribution of magnetic field. The actuator performance are compared under the sliding mode control algorithm and proportional–integral–derivative (PID) control algorithm. The parameters are compared with those of similar configurations.

## 2. Structure and Working Principle

**Figure 1:**
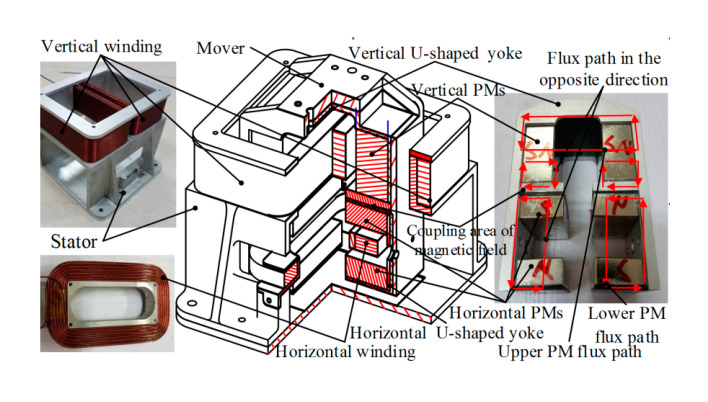
Construction of the 2-DOF Lorentz force actuator.

The structure of the novel 2-DOF Lorentz force actuator is presented in Figure 1. It is mainly composed of the wings, permanent magnets (PMs) and U-shaped yokes. The upper magnetic flux generated by vertical single layer PMs forms a closed circuit through the vertical U-shaped yoke and the lower magnetic flux produced by horizontal single layer PMs forms a closed circuit through the horizontal U-shaped yoke. Two vertical windings in the upper magnetic field produce vertical Ampere force and the horizontal winding in the lower magnetic field produces horizontal Ampere force, which means the 2-DOF Lorentz force actuator realizes the integration output of vertical driving force and horizontal driving force. To reduce the coupling between the upper flux and lower flux, a non-magnetic material partition is located between vertical PMs and horizontal PMs. And flux direction of upper vertical PMs and lower horizontal PMs are opposite in the longitudinal direction marked with red arrow in Figure 1. The overall dimension is 117 mm × 106 mm × 118 mm. The translation stroke is ±5 mm and the deflection angle is ±10°.

**Figure 2:**
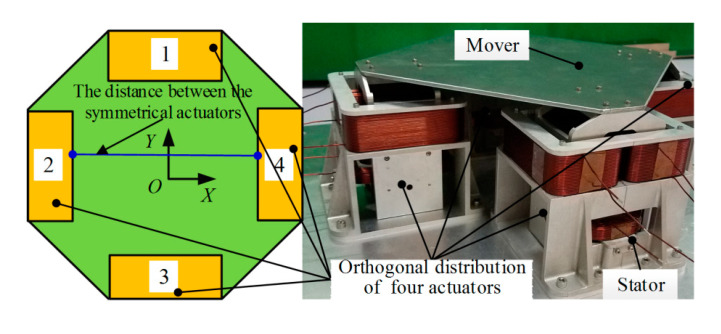
Magnetic suspension platform.

The structure of the modular magnetic suspension platform is shown in Figure 2. Four 2-DOF Lorentz force actuators are distributed orthogonal to each other. The mapping relationship between the motion direction of the platform and the driving force of the actuators is shown in Table 1. With the distance between the two actuators in the symmetrical position adjusted, the bearing capacity and motion range of the platform will be changed accordingly. So the performance of the modular magnetic suspension platform can be improved for varies applications.

The performance of the modular magnetic suspension platform depends on the 2-DOF Lorentz force actuator. It is necessary to study its characteristics of air-gap magnetic field and electromagnetic force.

## 3. Mathematical Model

### 3.1. Analysis of Magnetic Circuit

**Figure 3:**
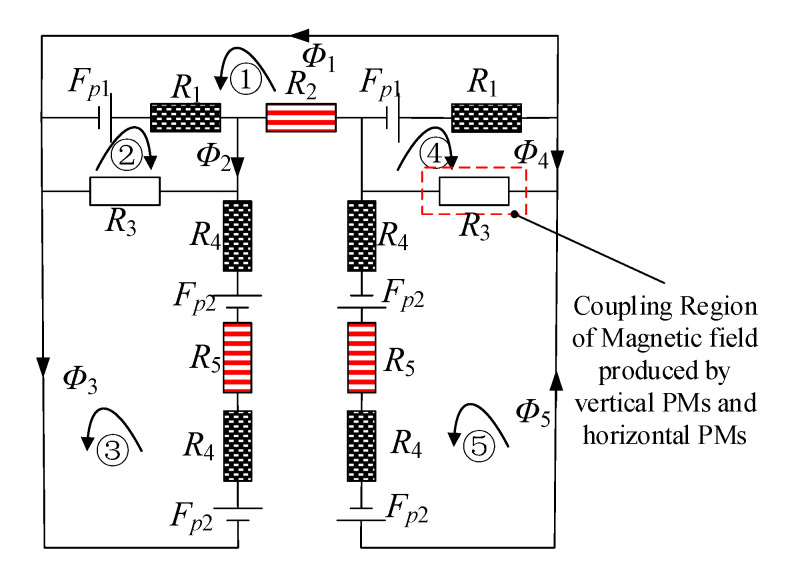
Equivalent magnetic circuit.

The equivalent magnetic circuit method is used to calculate magnetic field of the air-gap. The U-shaped yoke around the PMs contributes to reduction of the flux leakage, so the flux leakage is ignored for calculation convenience. Because the constant magnetic field is provided by PMs and the thickness of the yoke is large, the saturation phenomenon is ignored. The corresponding equivalent magnet circuit of the 2-DOF Lorentz force actuator can be obtained as shown in Figure 3. Circuit ① is upper flux path produced by the vertical PMs. Circuit ② is coupling flux path produced by vertical PMs and left horizontal PMs. Circuit ③ is left lower flux path produced by left horizontal PMs. Circuit ④ is flux path produced by vertical PMs and right horizontal PMs. Circuit ⑤ is right lower flux path produced by right horizontal PMs. *F_p_*_1_ and *F_p_*_2_ are magnetomotive force of vertical PMs and horizontal PMs, respectively. *R*_1_ and *R*_4_ are respectively internal resistance of PMs and horizontal PMs. *R*_2_ and *R*_5_ are respectively magnetic resistance of air-gap in the upper and the lower flux. *R*_3_ is the resistance of partition region where the upper flux and the lower flux are coupling. *Φ*_1_–*Φ*_5_ are the magnetic flux corresponding to circuits ①–⑤, respectively.

The magnetic circuit equation can be obtained by Kirchhoff law, as described by:

(1) $$\left[\begin{array}{ccc} 2R_{1}+R_{2} & 2R_{1} & 0\\ R_{1} & R_{1}+R_{3} & R_{3}\\ 0 & R_{3} & 2R_{4}+R_{3}+R_{5}\\ R_{1} & R_{1}+R_{3} & R_{3}\\ 0 & R_{3} & 2R_{4}+R_{3}+R_{5} \end{array}\right]\left[\begin{array}{c} \Phi_{1}\\ \Phi_{2}\\ \Phi_{3}\\ \Phi_{4}\\ \Phi_{5} \end{array}\right]=\left[\begin{array}{cc} \begin{array}{l} 2\\ 1\\ 0\\ 1\\ 2 \end{array} & \begin{array}{l} 0\\ 0\\ 2\\ 0\\ 0 \end{array} \end{array}\right]\left[\begin{array}{c} F_{p1}\\ F_{p2} \end{array}\right]$$

Circuits ② and ④, ③ and ⑤ are symmetrical respectively and there are *Φ*_2_ = *Φ*_4_, *Φ*_3_ = *Φ*_5_. Equation (1) can be simplified as:

(2) $$\left[\begin{array}{ccc} 2R_{1}+R_{2} & 2R_{1} & 0\\ R_{r1} & R_{1}+R_{3} & R_{3}\\ 0 & R_{3} & 2R_{4}+R_{3}+R_{5} \end{array}\right]\left[\begin{array}{c} \Phi_{1}\\ \Phi_{2}\\ \Phi_{3} \end{array}\right]=\left[\begin{array}{cc} \begin{array}{l} 2\\ 1\\ 0 \end{array} & \begin{array}{l} 0\\ 0\\ 2 \end{array} \end{array}\right]\left[\begin{array}{c} F_{p1}\\ F_{p2} \end{array}\right]$$

*F_p_*_1_ and *F_p_*_2_ can be expressed as:

(3) $$F_{pi}=H_{c}h_{i},$$

where *H_c_* is the permanent magnetic field intensity and *h_i_* is the thickness of the PMs in the magnetization. The reluctance *R*_1_–*R*_5_ can be expressed as:

(4) $$R_{i}=\frac{l_{i}}{u_{0}u_{i}s_{i}},$$

where *l_i_* is length of magnetic path at *R_i_* region. *μ*_0_ is the permeability of vacuum. *μ_i_* is the relative permeability. *s_i_* is section of magnetic path at *R_i_* region.

Substituting Equation (3) and Equation (4) into Equation (2), the magnetic flux density ***B*** can be calculated as:

(5) $$B={\left[B_{1}\;B_{2}\;B_{3}\right]}^{T}=diag{\left(A_{1},\;A_{2},\;A_{3}\right)}^{-1}{\left[\Phi_{1}\;\Phi_{2}\;\Phi_{3}\right]}^{T},$$

where *Φ*_1_, *Φ*_2_ and *Φ*_3_ are magnetic flux through *R*_1_, *R*_2_ and *R*_3_, respectively. *B*_1_, *B*_2_ and *B*_3_ are magnetic flux density at *R*_1_, *R*_2_ and *R*_3_, respectively. *A*_1_, *A*_2_ and *A*_3_ are effective areas corresponding to *Φ*_1_, *Φ*_2_ and *Φ*_3._

### 3.2. Calculation of Electromagnetic Force

The effective length of the vertical coil and the horizontal coil do not change when the mover deflects around the *x*-axis. When the mover deflects *α* around the *y*-axis, the effective length of the horizontal coil remains unchanged, while the effective length of the vertical coil increases. When the mover deflects *β* around the *z*-axis, the effective length of the vertical coil remains unchanged, while the effective length of the horizontal coil increases. Horizontal driving force *F_y_* and vertical driving force *F_z_* can be written as:

(6) $$\left\{ \begin{array}{l} F_{y}=N_{1}B_{4}I_{y}L_{1}\cos\beta =\frac{(4R_{1}R_{3}+4R_{1}R_{4}+2R_{1}R_{5}+4R_{3}R_{4}+2R_{3}R_{5})h_{1}-2R_{1}R_{3}h_{2}}{\left(2R_{1}+R_{2})(2R_{1}R_{3}+4R_{1}R_{4}+2R_{1}R_{5}+2R_{3}R_{4}+R_{3}R_{5}\right)}N_{1}L_{1}HI_{y}\cos\beta \\ F_{z}=N_{2}B_{1}I_{z}L_{2}\cos\alpha =\frac{R_{3}h_{1}+(4R_{1}+2R_{3})h_{2}}{\left(2R_{1}R_{3}+4R_{1}R_{4}+2R_{1}R_{5}+2R_{3}R_{4}+R_{3}R_{5}\right)}N_{2}L_{2}H_{c}I_{z}\cos\alpha \end{array}\right.$$

where *N*_1_ and *N*_2_ are numbers of horizontal windings and vertical windings, respectively_._ *I_y_* and *I_z_* are control current in the horizontal windings and vertical windings, respectively_._ *L*_1_ and *L*_2_ are the effective length of the initial state of the horizontal windings and vertical windings in the magnetic field, respectively.

It can be seen from Equation (6) that both horizontal electromagnetic force *F_y_* and vertical electromagnetic force *F_z_* are affected by the reluctance *R*_3_ where the upper flux and the lower flux are coupling. Horizontal electromagnetic force *F_y_* is affected by both the upper air-gap reluctance *R*_2_ and the lower air-gap reluctance *R*_5_. The vertical electromagnetic force *F_z_* is mainly affected by the lower air-gap reluctance *R*_5_. So there are obvious coupling between the upper flux and the lower flux in the 2-DOF Lorentz force actuator.

## 4. Mathematical Model Analysis of Distribution Characteristics of Magnetic Flux Density

The performance of electromagnetic force of the 2-DOF Lorentz force actuator mainly depends on distribution of the air-gap magnetic field. So the magnetic field characteristics of the actuator are analyzed in this section.

**Figure 4:**
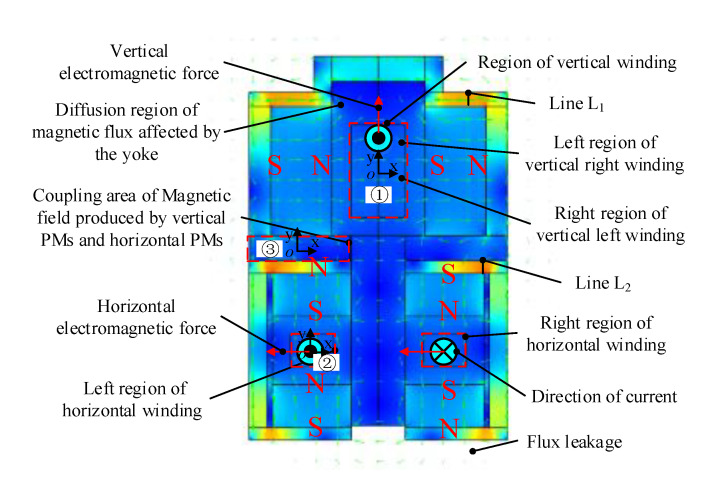
Flux nephogram of the 2-DOF Lorentz force actuator.

Combined with the performance requirements and overall size constraints of the magnetic suspension platform in practical applications, the nonlinear model of the Lorentz force actuator size optimization was calculated by the Optimization Toolbox in MATLAB to reduce weight and energy consumption. The optimized parameters of the Lorentz force actuator are listed in Table 2. The mass of the actuator is 2.59 kg, the translation stroke is ±5 mm, the overall dimension is 117 mm × 106 mm × 118 mm and the deflection angle is ±10°. The PM material is NdFeB. Magnetic energy product (BH product) is about 358 KJ/m^3^. Residual magnetism is about 1.36 T. Coercive force is about 1026 KA/m and working temperature can reach 120 °C. The material of the yoke is 1J22 with high saturation magnetic induction (about 0.245 T). The flux nephogram at the central section is shown in Figure 4. To reduce magnetic flux leakage and increase magnetic field of air-gap, magnetic flux of the upper PMs and the lower PMs form a closed magnetic circuit through the yoke, respectively. However, there is obvious flux diffusion at the region near the yoke marked with black ellipses in Figure 4, which makes the magnetic flied inhomogeneous.

The magnetic flux density in the vertical winding region ① and the left horizontal winding region ② are analyzed. The coupling of the upper flux and the lower flux in the partition region ③ is analyzed.

**Figure 5:**
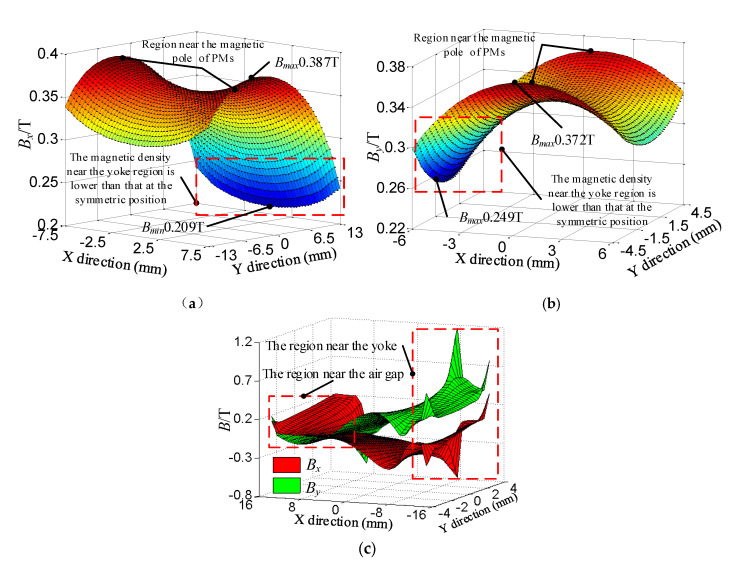
Magnetic field distribution of the 2-DOF Lorentz force actuator: (**a**) Distribution of magnetic flux density at the vertical winding region ①; (**b**) distribution of magnetic flux density at the left horizontal winding region ②; (**c**) distribution of magnetic flux density at the partition region ③.

In the vertical winding region ①, the magnetic flux density is large in the middle of the air-gap and decreases along both ends in the Y direction. The magnetic flux density is small in the middle of the air-gap and increases along both sides in the X direction, as shown in Figure 5a. Because the vertical yoke leads to a large gradient of magnetic flux intensity in the upper end of the vertical winding, the magnetic flux density near the yoke area is lower than that at the symmetric position. The magnetic permeability of the yoke is greater than that of the air, the magnetic flux generated by the upper edge of the vertical PMs pass through the yoke preferentially. So a few magnetic flux pass through the upper end area of the vertical winding. The minimum value of 0.209 T is at the center of the top of the upper winding. The maximum value of 0.387 T is in the middle position of the edge of the upper winding. The average of the magnetic flux density is 0.333 T.

Due to the similar configuration of magnetic flux circuit, the magnetic flux density distribution at the left horizontal winding region ② is similar to that of vertical winding region ①, as shown in Figure 5b. The magnetic flux density decreases rapidly near the yoke, resulting in a large difference between the maximum value of 0.372 T and minimum value of 0.249 T. The average of the magnetic flux density is 0.332 T.

It can be seen from Equations (6) and (4) that the upper flux and the lower flux are coupled in the partition region. The vertical and horizontal components of magnetic flux density in the partition region ③ are calculated, as shown in Figure 5c. The horizontal component *B_x_* and the vertical component *B_y_* are large, which indicates the coupling of upper flux and lower flux is strong. That is because vertical PMs and horizontal PMs are close and U-shaped yoke affects magnetic flux density distribution. The coupling of upper flux and lower flux is strong in the region near the yoke and is weak in the region near the air-gap. So the yoke is helpful to reducing magnetic flux leakage and increasing air-gap magnetic field strength. But the magnetic field gradient is large and the magnetic density uniformity is poor at the region near the yoke.

**Figure 6:**
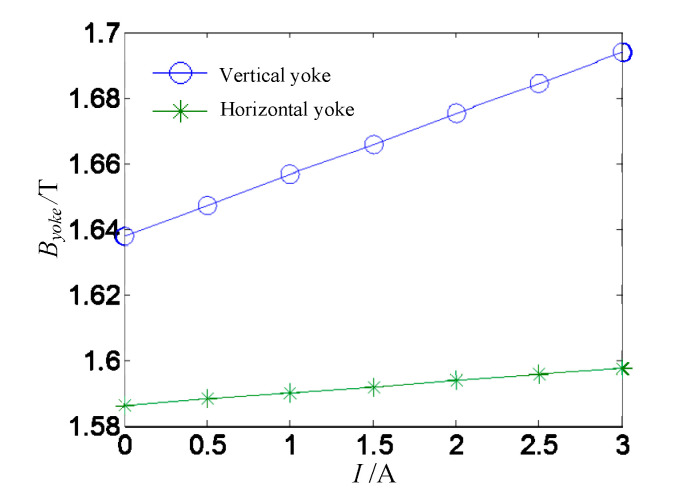
Magnetic flux density of the yoke versus the excited current.

The Line L_1_ and the Line L_2_ are respectively on the maximum magnetic density region of the vertical yoke and the horizontal yoke in Figure 4 and their magnetic flux density curves versus the excited current are plotted in Figure 6. The magnetic flux density of the vertical yoke increases linearly with the increase of excited current. That’s because the coil wound on the vertical yoke produces a large electromagnetic field and the permanent magnetic field produced by the PM is superimposed in the vertical yoke. When the excited current is 3 A, the maximum flux density in the vertical yoke is 1.694 T, which is less than 2.45 T of saturation flux density. Because the number of turns of the horizontal coil is small and it is not around the yoke, the electromagnetic field generated by the coil is low and the maximum magnetic density in the horizontal yoke is 1.598 T. The maximum magnetic flux density in the vertical yoke and the horizontal yoke is not saturated and the magnetic flux density fluctuation is 3.4% and 0.71%, respectively.

## 5. Experimental Verification

### 5.1. Characteristics of Electromagnetic Force

The magnetic field distribution in the critical region is analyzed above. The driving performance of the actuator is directly reflected in the characteristics of the electromagnetic force. The driving forces are analyzed by 3D FEM and experimental measurement.

**Figure 7:**
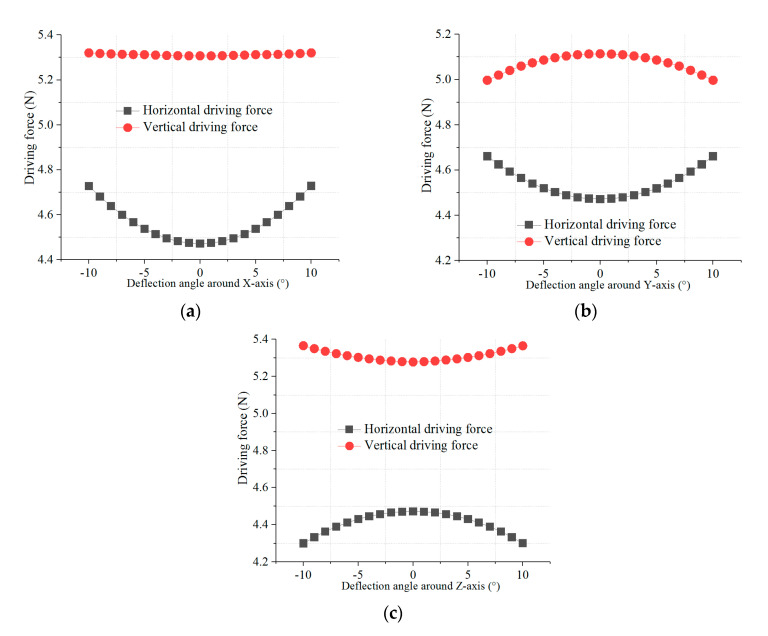
Driving force versus deflection: (**a**) Deflection around *X*-axis; (**b**) deflection around *Y*-axis; (**c**) deflection around *Z*-axis.

It is difficult to directly measure the moment of the actuator, so FEM is used to analyze the driving force when the mover deflects around the *x*-axis, *y*-axis and *z*-axis, respectively. As shown in Figure 7, the vertical driving force is larger than the horizontal driving force, because magnetic flux density in the vertical winding region is larger than that in the horizontal winding region and turns of the vertical winding are more than that of the horizontal winding. As shown in Figure 7a, the driving forces increase when the mover deflects around the *x*-axis. That is because the windings approach the surface of the pole of PMs and the magnetic flux density increases, as shown in Figure 5a. The change rates of the vertical driving force and the horizontal driving force are 1.5% and 4.5%, respectively.

As shown in Figure 7b, the horizontal driving force increases gradually when the mover deflects around the *y*-axis, because the horizontal winding is close to the surface of the magnetic pole and magnetic flux density increases. The vertical driving force gradually reduces, because the effective length of the vertical coils is just a small amount of increase and the magnetic flux density rapidly reduces. The change rates of the vertical driving force and the horizontal driving force are 4.1% and 2.3%, respectively. 

As shown in Figure 7c, the horizontal driving force rapidly reduces when the mover deflects around the *z*-axis, because the horizontal winding is close to the surface of yoke and magnetic flux density reduce, as shown in Figure 5b. The vertical driving force increases, because the vertical winding is close to the magnetic pole and magnetic flux density increase, as shown in Figure 5a. The change rates of the horizontal driving force and the vertical driving force are 3.8% and 1.6%, respectively.

Based on the above analysis, the change of the effective length of the coil and the inhomogeneity of air-gap magnetic flux density make the driving force fluctuate obvious. The linearity of the driving force is more affected by the uneven of the magnetic flux density.

**Figure 8:**
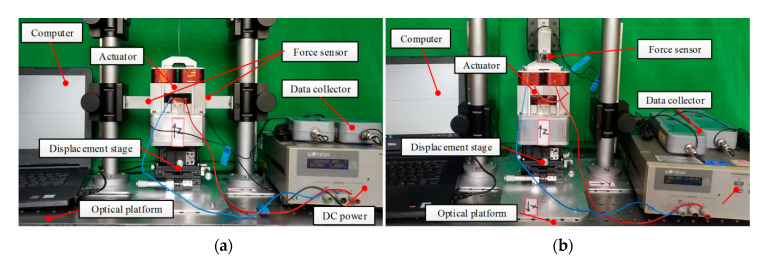
Experiment platform: (**a**) Measurement of horizontal driving force; (**b**) measurement of vertical driving force.

Driving force variation with control current and displacement are studied by FEM and experimental measurements, respectively. To reduce the manufacturing error and assembly error, differential measurement method is adopted in the experiment, as shown in Figure 8. To compensate the weight of the mover, the actuator is suspended through the thread. The three-axis displacement table is adjusted so that the winding is at the zero position, as shown in Figure 8a. The driving force in vertical direction is measured as shown in Figure 8b.

**Figure 9:**
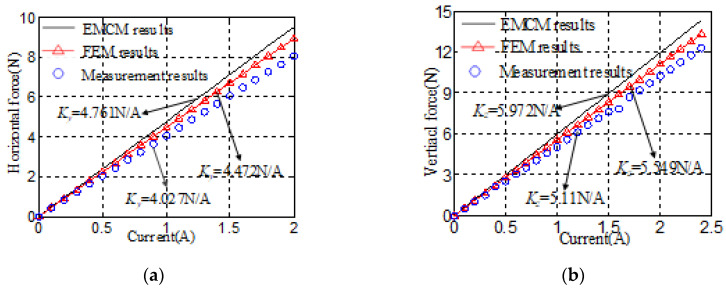
Driving force versus control current: (**a**) horizontal driving force; (**b**) vertical driving force.

Driving force variation with control current is shown in Figure 9. With the increase of current, the electromagnetic force increases linearly. Due to ignorance of the magnetic flux leakage in the calculation process, EMCM results of horizontal current stiffness and vertical current stiffness, which are 4.761 N/A and 5.972 N/A, both are larger than FEM results and experimental results, respectively. The error between FEM results and experimental results increases as the control current increases. There are some threaded holes on the yoke for processing and assembly, which inevitably increases the magnetic resistance and reduces the magnetic flux density in air-gap. The magnetic resistance of threaded holes is ignored by FEM. So the FEM results are larger than the experimental results. FEM results of the vertical force coefficient and the horizontal force coefficient are 4.472 N/A and 5.549 N/A, respectively. Compared to experimental results of 4.08 N/A and 5.11 N/A, the error is 9.95% and 8.59%, respectively. 

**Figure 10:**
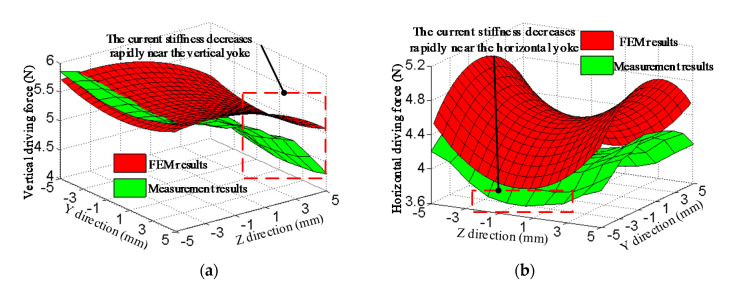
Results of finite-element method (FEM) and measurement of the driving force: (**a**) Vertical driving force; (**b**) horizontal driving force.

With the control current of 1 A, driving force variation with displacement is analyzed by FEM and experimental measurement. The results of FEM and measurement are shown in Figure 10. FEM results are slightly higher the experimental results, because the magnetic resistance of threaded holes is ignored by FEM. The variation trend of driving force is consistent with that of magnetic field. The driving force decreases faster at the region near the yoke, because the magnetic field gradient near the yoke is large and the magnetic flux density decreases rapidly. The maximum of driving force of FEM is 5.76 N, which is smaller than 5.85 N of experiment results. The minimum of the vertical driving force of FEM is 4.86 N, which is larger than 4.17 N of experiment results. FEM value of average vertical driving force is 5.42 N. Compared to the experiment value of 5.19 N, the error is 4.2%. The maximum horizontal value of FEM is 5.03 N, which is larger than 4.44 N of experiment results. The minimum horizontal current stiffness of FEM is 3.92 N, which is larger than 3.66 N of experiment results. FEM value of average horizontal driving force is 4.48 N. Compared to the experiment value of 4.08 N, the error is 8.9%.

**Figure 11:**
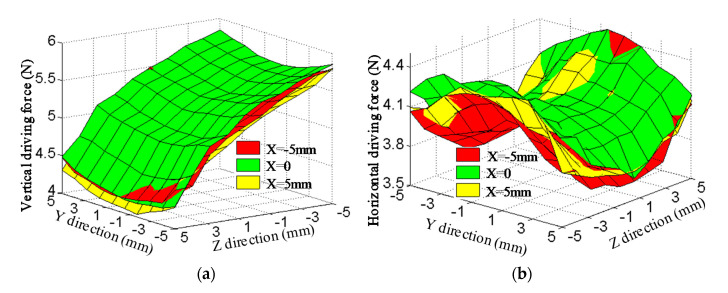
Experimental results of driving force: (**a**) Vertical driving force; (**b**) horizontal diving force.

With the control current of 1 A, driving force variation with displacement in full range of motion is analyzed through experimental measurement, as shown in Figure 11. The maximum and minimum of the vertical driving force are 5.85 N and 4.04 N, respectively. The fluctuation of the vertical driving force is 30.9%. The maximum and minimum of the horizontal driving force are 4.48 N and 3.51 N, respectively. The fluctuation of the horizontal driving force is 21.6%. The reason for the large fluctuation is the non-uniformity of the air-gap magnetic field. 

Large horizontal driving force and vertical driving force output is implemented simultaneously by the 2-DOF Lorentz force actuator. The distribution law of air-gap magnetic field is similar to that of driving force. So the linearity of driving force can be improved by heightening the uniformity of air-gap magnetic field.

### 5.2. Levitation Performance

**Figure 12:**
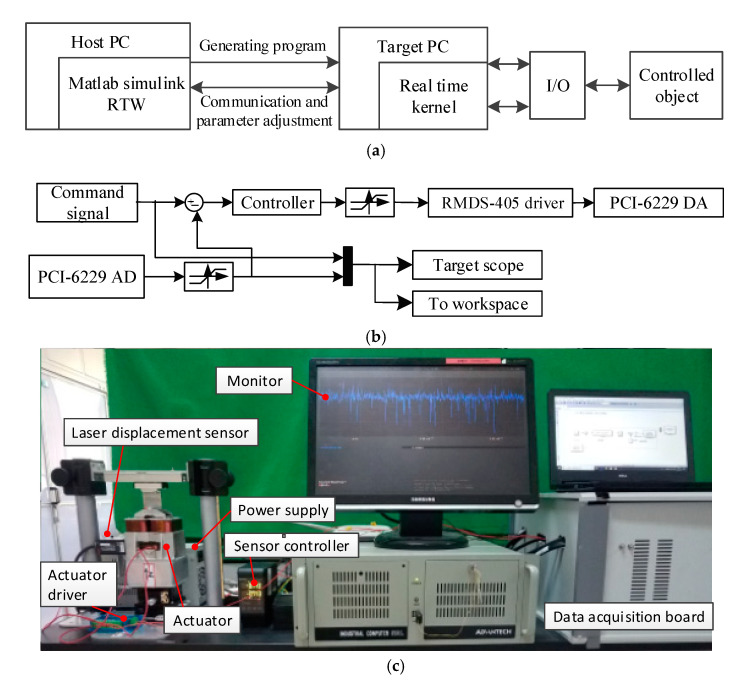
Experimental system: (**a**) Simulink Real-Time hardware; (**b**) Close-loop model; (**c**) test system.

The control system of the actuator is established through the Simulink Real-Time hardware in the loop simulation system, as shown in Figure 12a. The control program is established under the MATLAB/Simulink environment of the host computer. The control model is shown in Figure 12b. The channel of the PCI-6229 DA is used to output the control voltage of the RMDS-405 driver. The channel of the PCI-6229 AD collects the analog voltage value output by the laser displacement sensor. The test system is shown in Figure 12c. The position sensors are laser displacement sensor ILD2300 with a resolution of 1 µm. The command signals are step, sine and continuous step to verify the suspension performance of the actuator.

**Figure 13:**
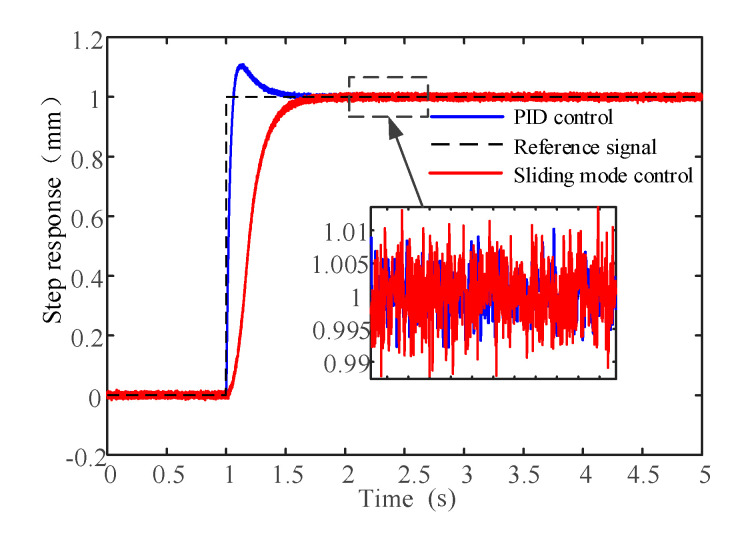
Test results with the step response.

The step response is set at 1 mm and the sampling frequency is 2 KHz. As shown in Figure 13, it can be seen that the system can reach the command signal within a limited time range under the two control algorithms. The system adjustment time under PID control algorithm is 0.38s and the overshoot is 10.8%. Under the sliding mode control algorithm, the system adjustment time is 0.58s and the system has no overshoot.

**Figure 14:**
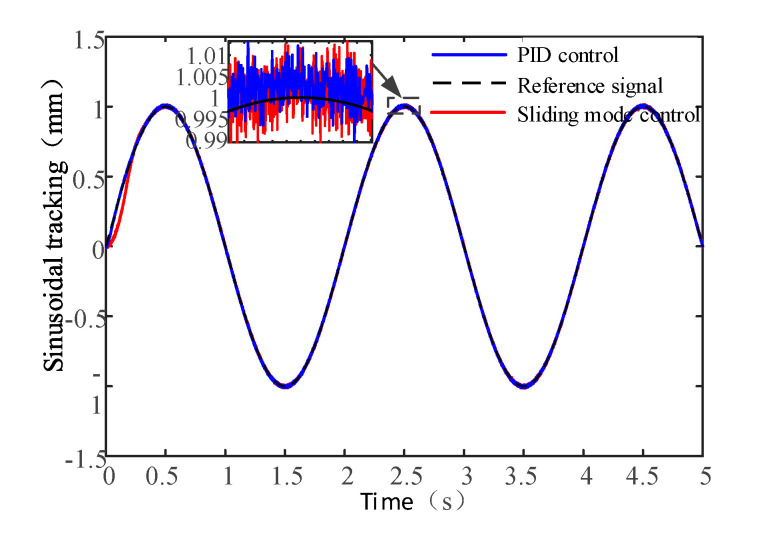
Test results with the sinusoidal signal.

Test results with the sinusoidal signal are shown in Figure 14. When the input signal frequency is 0.5 Hz, it can be seen that good tracking performance is achieved with two different control algorithm. The closed-loop control system with PID control algorithm has faster response speed and achieves stable tracking in a short time. The system with sliding mode control algorithm takes more time for stable tracking.

**Figure 15:**
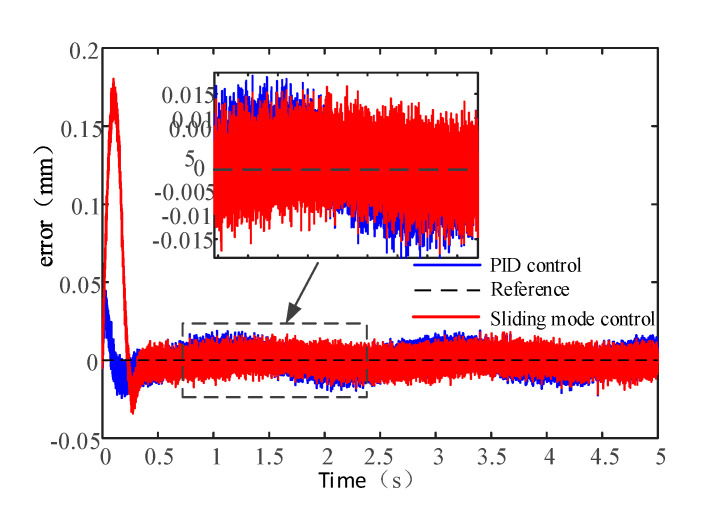
Sine signal tracking error.

As shown in Figure 15, the tracking error roughly takes on the shape of a sine by PID control algorithm. The error under the sliding mode control algorithm floats around 0 when the system is stable and the maximum error is less than that under PID control algorithm. The tracking accuracy of the sliding mode control algorithm is better than that of the PID control algorithm.

**Figure 16:**
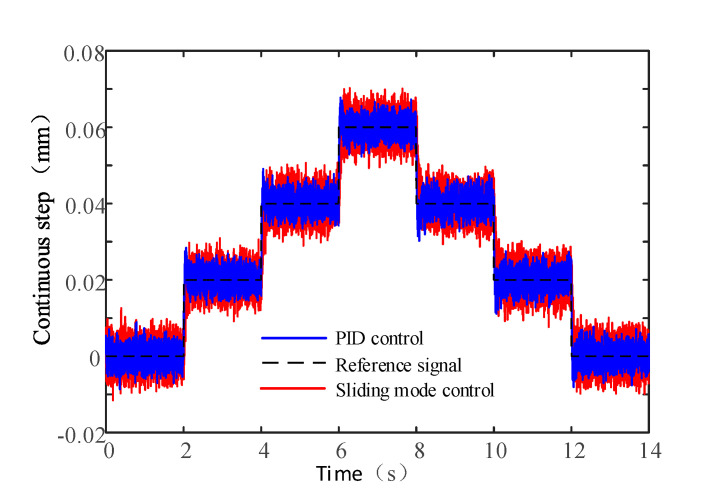
Positioning accuracy.

The positioning accuracy of the actuator is tested with a 0.02 mm continuous step command signal. The positioning accuracy not only depends on the air gap magnetic field characteristics but also on the sensor detection accuracy, resolution, motion control board, control algorithm and other factors. The data acquisition board is NI PCI −6229 and the sampling frequency is 2 KHz. As shown in Figure 16, the positioning accuracy of the actuator with sliding mode control algorithm and PID control algorithm are about 20 μm and 15 μm, respectively.

The 2-DOF Lorentz force actuator developed in this paper is similar to that in Reference [35]. The structure parameters and performance are compared, as shown in the Table 3. The motion range of the Lorentz force actuator in this paper is larger, especially the deflection angle is up to ±10°, which is 2.5 times of the reference value. The larger air gap makes the uniformity of magnetic density poor and the positioning accuracy is only 15 μm, which is lower than 4.4 nm in the reference. Due to the large size of the PM and air gap, the air gap flux density of 0.33 T is close to that of 0.34 T in the reference. Because of the large size of the coil in the magnetic field, vertical force coefficient of 5.549 N/A and horizontal force coefficient of 4.027 N/A are larger than those of 1.8 N/A and 0.31 N/A in the reference, respectively. So Lorentz force actuator in this paper effectively increases the deflection angle and force coefficient at the cost of localization accuracy.

## 6. Conclusions

Considering the pose of the windings and the PMs, a novel 2-DOF Lorentz force actuator is developed for the modular magnetic suspension platform. The mass of the actuator is 2.59 kg, the translation stroke is ±5 mm, the overall dimension is 117 mm × 106 mm × 118 mm and the deflection angle is ± 10°. The mathematical model of electromagnetic force is established by equivalent magnetic circuit method. The characteristics of magnetic field and the driving force are analyzed through the FEM and the experimental measurement. The horizontal force coefficient and the vertical force coefficient are 4.08 N/A and 5.11 N/A, which means large force output of the actuator is realized. The sliding control algorithm and PID control algorithm are respectively applied to the actuator control system. The experimental results show that the position precision under the PID control is 15 μm, which is better than 20 μm of sliding model control. The tracking accuracy of the sliding mode control algorithm is better than that of the PID control algorithm. Compared with the conventional similar 2-DOF Lorentz force actuator, the motion range and force coefficient of the actuator in this paper are larger. Due to the increase of the air gap size, the uniformity of air gap flux density and positioning accuracy are not as good as those of small sized actuator. The results of the paper provide important support for the design of the high performance 2-DOF Lorentz force actuator. The design method of magnetic flux decoupling will be studied in the future.

## Figures and Tables

**Table 1:** The relationship between motion direction of the platform and driving forces of the actuators.

Movement of the Platform	Driving Force
Translation along *X*-axis direction	Horizontal driving forces produced by actuators 1and 3
Translation along *Y*-axis direction	Horizontal driving forces produced by actuators 2 and 4
Translation motion in *Z*-axis direction	Vertical driving forces produced by actuators 1, 2, 3 and 4
Deflection around *X*-axis	Vertical driving forces produced by actuators 1 and 3
Deflection around *Y*-axis	Vertical driving forces produced by actuators 2 and 4
Rotate around *Z*-axis	Horizontal driving forces produced by actuators 1, 2, 3 and 4

**Table 2:** The structural parameters of the actuator.

Parameter	Value
Length of the vertical PM/mm	36
Width of the vertical PM/mm	45
Thickness of the vertical PM/mm	17
Distance between vertical PM and horizontal PM/mm	7
Layer number of the vertical winding	26
Turns of the vertical winding	364
Air-gap between vertical PMs/mm	25
Air-gap between horizontal PMs/mm	19
Length of the horizontal PM/mm	22
Width of the horizontal PM/mm	45
thickness of the horizontal PM/mm	14
Turns of the horizontal winding	108
Layer number of horizontal winding	9
Radius the coil/mm	0.5
Weight of the actuator/kg	2.59
The overall dimension/mm	117 × 106 × 118

**Table 3:** The parameters of the actuator in the paper and the reference.

Parameters	In this Paper	In the Reference
Wire diameter	1 mm	0.41 mm
Vertical coils	2 × 182 turns	2 × 130 turns
Horizontal coils	108 turns	2 × 22 turns
Maximum continuous current	2 A	0.9 A
Magnetic flux density	0.33 T	0.34 T
Vertical force coefficient	5.549 N/A	1.8 N/A
Horizontal force coefficient	4.027 N/A	0.31N/A
Working range	5 × 5 × 5 mm	2.5 × 2.5 × 2.5 mm
Rotation	10° × 10°×10°	4° × 4°× 4°
Resolution	15 μm	Better than 4.4 nm
